# Genetics of Alzheimer’s Disease

**DOI:** 10.1007/s13311-014-0295-9

**Published:** 2014-08-12

**Authors:** Rita Guerreiro, John Hardy

**Affiliations:** Department of Molecular Neuroscience, Institute of Neurology, UCL, 1 Wakefield Street, London, WC1N 1PJ UK

**Keywords:** Alzheimer's disease, Genetics, Next generation sequencing, Genome-wide association studies

## Abstract

The analyses of genetic factors contributing to Alzheimer’s disease (AD) and other dementias have evolved at the same pace as genetic and genomic technologies are developed and improved. The identification of the first genes involved in AD arose from family-based studies, but risk factors have mainly been identified by studies comparing groups of patients with groups of controls. The best outcomes have been heavily associated with the capacity of interrogating genetic variability at the genome level without any particular a priori hypothesis. In this review we assess the role of genetic family studies in Alzheimer’s disease and other dementias within the current status of dementias’ and, particularly, AD’s genetic architecture.

**Electronic supplementary material:**

The online version of this article (doi:10.1007/s13311-014-0295-9) contains supplementary material, which is available to authorized users.

## Introduction

Linkage analyses performed in the early 1990s and centered on early onset Alzheimer’s disease families identified the only fully penetrant mutations known to date to be involved in this disease. Three genes were found to carry these mutations: *APP*, *PSEN1* and *PSEN2* (Amyloid beta (A4) precursor protein, Presenilin 1, and Presenilin 2). Additionally, a strong risk factor was also identified through family studies: the E4 allele of *APOE* (Apolipoprotein E) was found to increase the risk for Alzheimer’s disease in different populations [1–4].

After these findings no gene was conclusively associated with Alzheimer’s disease for almost 15 years.

Traditional approaches to the study of Mendelian diseases relied on genetic linkage studies (a powerful tool to detect the chromosomal location of disease genes which is based on the observation that genes that reside physically close on a chromosome remain linked during meiosis). These studies involved large multi-generational pedigrees and needed the availability of multiple affected and unaffected individuals for testing. [5]. When such families were available, genetic markers would be used to determine which areas of the genome only the affected individuals shared. This would provide evidence regarding the location of the causative gene, after which, DNA sequencing, usually be means of the Sanger method (based on the selective incorporation of chain-terminating dideoxynucleotides by DNA polymerase during in vitro DNA replication) would be performed to pinpoint the actual mutation. Although a powerful approach, that has yielded many substantive findings, genetic linkage studies have a number of drawbacks that limit its utility: 1) large pedigrees are, in many cases, not available for study, particularly for late-onset diseases, where older generations have often died and descendants have not yet reached the age at onset; 2) the markers used to perform linkage, although dispersed throughout the genome, were usually in the hundreds, meaning that regions of linkage were very extensive and generally containing tens to hundreds of genes, having a clear impact on the costs of follow-up by sequencing; 3) the amount of time required to study a single family using this approach was quite significant, usually several months to years [6].

More recently, the development of genome-wide genotyping and second-generation sequencing technologies has drastically changed the approaches used to study Mendelian disorders. Diseases presenting an autosomal recessive pattern of inheritance are well suited for autozygosity analysis (performed by high-density whole genome genotyping) and whole exome sequencing. In some cases, diseases presenting an autosomal dominant pattern of inheritance can now be studied by analyzing the exome variability in one or two affected family members without the need for linkage analyses. Although linkage analyses will be valuable and in some cases essential, isolated exome sequencing analyses in small families with neurological diseases already resulted in many significant findings.

## Genome-Wide Association Studies (GWAS)

Over 20 genetic loci with low risk effects for Alzheimer’s disease have been identified by GWAS [7] (Table 1). These studies assess common genetic variability at the genome level between large groups of cases and controls. Each of the loci identified contributes modestly to the risk of developing the disease, but new polygenic models are currently being investigated to try and establish more accurately who will develop Alzheimer’s disease. GWAS were not designed to identify genes or variants. This approach was rather designed to find loci fitting the common disease – common variant hypothesis of human disease (for a description of GWAS in human disease refer to [8]) and the identification of the specific variant(s) and true effect(s) on the development of AD can only be achieved with fine mapping and functional analyses of each locus.

**Table 1:** Most significant associations found by GWAS in the recent IGAP (International Genomics of Alzheimer’s Project) meta-analysis.

Loci		Odds ratio (OR)	Possible biological pathways
*BIN1*	Bridging integrator 1	1.22	Endocytosis
*PICALM*	Phosphatidylinositol binding clathrin assembly protein	0.87	Endocytosis
*CLU*	Clusterin	0.86	Immune and complement systems/inflammatory response; cholesterol/lipid metabolism
*CR1*	Complement component (3b/4b) receptor 1 (Knops blood group)	1.18	Immune and complement systems/inflammatory response
*MS4A6A*	Membrane-spanning 4-domains, subfamily A, member 6A	0.90	Immune and complement systems/inflammatory response
*ABCA7*	ATP-binding cassette, sub-family A (ABC1), member 7	1.15	Immune and complement systems/inflammatory response; cholesterol/lipid metabolism
*SORL1*	Sortilin-related receptor, L(DLR class) A repeats containing	0.77	Endocytosis; lipid transport
*PTK2B*	Protein tyrosine kinase 2 beta	1.10	
*EPHA1*	EPH receptor A1	0.90	Immune and complement systems/inflammatory response; cholesterol/lipid metabolism
*HLA-DRB5-HLA-DRB1*	Major histocompatibility Complex, class II, DR beta 5 and DR beta 1	1.11	Immune and complement systems/inflammatory response; cholesterol/lipid metabolism

Ten most significant associations identified in the overall meta-analysis performed by the IGAP [14]. Loci are shown from the most to the least significant and ORs result from the overall meta-analysis of both GWAS stages, calculated for the minor alleles. The loci are represented by the genes thought to most probably have a role in AD pathogenesis as part of the biological pathways indicated in the right column [9].

It is also important to note that these loci seem to be part of a few biological pathways, indicating that these pathways, although it is currently not clear how, have an essential role in the pathobiological processes occurring in Alzheimer’s disease. Three main pathways can be identified: the immune system and inflammatory responses; cholesterol and lipid metabolism; and endosomal vesicle recycling [7, 9]. All these pathways have previously been associated with AD. For instance, neuropathological analyses of AD brains and functional assays connecting the amyloid beta protein to the complement pathway activation have given consistent evidence of the involvement of several complement cascade factors in the pathogenesis of this disease [10, 11]. Another example is SORL1 (sortilin-related receptor with A-type repeats), which has been extensively studied in AD due to its role in APP metabolism [12]. SORL1 modulation of the APP metabolism partially occurs in the endosomal pathway as part of a unique regulatory pathway for the control of neuronal protein transport [13]. However, it has not been possible to undoubtedly associate variability in this gene to AD before the application of GWAS to the study of late onset Alzheimer’s disease (LOAD - Alzheimer’s disease occurring after 65 years of age). Over 15 case-control association studies of *SORL1* have produced positive, negative and trend associations between genetic variability at this gene and the risk of AD in different populations (Alzgene assessed on April 2014: http://goo.gl/xSRRl6). The initial GWAS did not show a significant association between AD and the *SORL1* locus. A significant association was only established in the recent meta-analysis using data from more than 70,000 people [14]. The contradictory initial case-control reports may have been caused by different inclusion criteria used, with only cohorts tightly controlled for ethnicity achieving significance [15]. Also important to substantiate the association of this gene with AD was the identification of potentially pathogenic mutations in French early-onset AD (EOAD – Alzheimer’s disease occurring before 65 years of age) cases. In this study the authors performed exome sequencing in 14 autosomal dominant EOAD index cases without mutations on the known AD genes and identified *SORL1* mutations in 5 patients (1 nonsense mutation and 4 missense), which were not found in healthy controls. In a replication sample, including 15 autosomal dominant EOAD cases, they identified 2 other novel mutations resulting in a total of 7 mutations in 29 cases [16]. Although independent replications and segregation analyses are still needed, these results indicate a potential pathogenic causative effect of *SORL1* mutations in AD.

## What is the Role of Family Studies in the Current Genomics Era?

The ability to sequence the whole genome, or more frequently, the whole exome of patients has led to a renewed interest in the study of Mendelian forms of diseases and family studies. Consequently, the potential contribution of rare genetic variants with strong effects in complex phenotypes like Alzheimer’s disease is currently under analysis.

These technologies, when applied to a large enough number of cases and controls, also have the potential to identify moderate risk factors with lower allele frequencies in the population than the ones identified by GWAS [17].

Variants with rare/moderate allele frequencies usually confer a greater risk for disease when compared with common variants due to purifying selection: high-risk alleles contributing to early-onset diseases may reduce reproductive fitness and consequently be driven to low frequencies [18]. Even though high risk alleles are rare in the population, these can be very informative by implicating genes with strong etiologic and biological roles in disease. These genes are usually the basis for functional genetic studies and considered important targets for the development of novel therapies [19, 20].

Extended families are an extremely valuable asset for genetic studies since the same chromosomal segments recur in relatives. However, when studying late onset disorders it is frequently difficult to identify such families and by studying a large number of these families it seems more and more plausible that no other fully penetrant dominant genes like *APP* and the *PSENs* genes are to be found in Alzheimer’s disease. Some of these families will likely have private mutations as the cause of their pathological processes.

## Contribution of Recessive Alleles to Alzheimer’s Disease

**Fig. 1:**
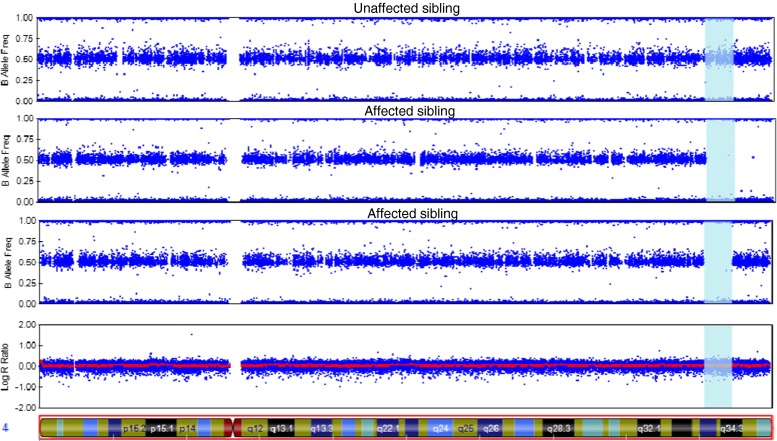
Homozygosity analysis in a family using SNP arrays

By studying rare recessive diseases we have recently been able to uncover significant genetic factors involved in common disorders like Alzheimer’s and Parkinson’s diseases [21, 22]. By performing autozygosity and exome sequencing analyses in Turkish dementia families we identified *TREM2* homozygous mutations causing frontotemporal dementia [23]. *TREM2* had previously been associated with Nasu-Hakola disease, a rare autosomal recessive form of dementia presenting with bone cysts [24]. Inbred (and frequently isolated) populations are ideal for the study of disease genetics. Inbreeding can increase the incidence of rare recessive disorders and isolated populations have an allelic load mostly derived from the alleles present in the population founders. Rare recessive alleles with an effect in disease risk or causation tend to be located within long genomic regions of homozygosity [20]. These long stretches of loss of heterozygosity can be easily assessed using SNP arrays (Fig. 1).

This figure represents the results from an Illumina Infinium whole-genome genotyping assay as visualized in GenomeStudio. It shows homozygosity mapping for three siblings (two affected by dementia and one unaffected). Each blue dot represents one individual marker or single-nucleotide polymorphism (SNP). For each SNP, a low B allele frequency indicates that the individual is a homozygote for the A allele; intermediate values mean they are a heterozygote and high B allele frequency means that they are a homozygote for the B allele. This example shows a region of chromosome 4 (vertical blue area) in which the affected siblings present a large (over 7 Mb) homozygous region that is not shared by the unaffected family member. This finding suggests the possibility that a disease-causing homozygous mutation may be present in this genomic region.

This strategy combining autozygosity analysis with exome sequencing allowed us to identify *TREM2* mutations in cases presenting with an atypical phenotype to that previously associated with the gene. The 3 cases we identified by exome sequencing would have never had the *TREM2* gene sequenced by standard methods because of the atypical presentation. This clearly represents the power of whole-exome and whole genome sequencing in the establishment of molecular diagnoses. The identification of mutations in genes previously associated with different phenotypes has been a consistent finding from the application of exome sequencing to neurological disorders. These results are critical to the understanding of the biological processes underlying these diseases by uncovering shared pathways [25–27].

Although LOAD does not exhibit obvious recessive inheritance, recessive alleles may play a role as genetic risk components in this form of the disease. An excess of runs of homozygosity and larger homozygous tracts have been reported in LOAD cases when compared to controls [28, 29]. In early-onset familial AD one mutation in *APP* (p.A673V) has been reported to cause disease only in the homozygous state, whereas heterozygous carriers are unaffected, which is consistent with a recessive Mendelian trait of inheritance [30]. The heritability for LOAD is estimated to be between 62 and 81 % and for EOAD between 92 and 100 %. Dominant causing mutations do not account for all this heritability and the belief that only highly penetrant dominant AD-causing mutations are in the basis of EOAD is inconsistent with epidemiologic data that found apparent autosomal dominant transmission in approximately only 10 % of all EOAD cases, leaving the majority of EOAD unexplained [31].

We have previously used whole-genome genotyping arrays to study two siblings from an Israeli family consisting of seven siblings from a first-cousin marriage. At the time of the clinical study the mother was 90 years old with mild gait difficulties but otherwise healthy, and particularly presenting no cognitive loss. The father died at age 90 due to cerebral stroke. All the other siblings were neurologically healthy. In this family we catalogued all the large regions of homozygosity shared between the two affected siblings and crossed this information with the genes known to have a possible role in AD [32]. We are currently applying exome sequencing to follow up on these results and pinpoint the mutation causing AD in this family. Other AD families presenting with apparent autosomal recessive patterns of inheritance are starting to be described in the literature and we expect the study of recessive alleles in AD to have significant results in the near future [33].

## TREM2 in Alzheimer’s Disease

Following from the fact that our initial application of exome sequencing to the study of neurological diseases consistently identified mutations in genes previously associated with different phenotypes. And particularly after the identification of *TREM2* homozygous mutations causing frontotemporal dementia, we extended the genetic analyses of this gene to other forms of dementia. Interestingly, when studying AD cases and controls we identified a heterozygous rare variant (p.R47H) that was significantly associated with an increased risk (OR > 3) for the development of AD [22]. This was the first gene to be identified with a moderate risk effect on the disease since the association of the E4 allele of APOE was established for AD [22, 34].

Taken together these results clearly show that the study of recessive forms of rare neurological diseases can not only have a direct impact in the families where mutations are found, but can also have an impact on more common forms of diseases by contributing to the identification of risk alleles and, consequently, to a greater understanding of the pathobiological pathways underlying these disorders.

TREM2 is a transmembrane glycoprotein, consisting of an extracellular immunoglobulin-like domain, a transmembrane domain, and a cytoplasmic tail, which associates with DAP12, also known as TYROBP. The association between these two proteins is required for its signaling function and it triggers the activation of immune responses in different immune cells [24]. TREM2 is known to control two streams of signaling to regulate the reactive microglial phenotype.

One of these streams regulates phagocytosis and is associated with enhanced phagocytic pathways (which could be relevant to the removal of cell debris and the clearance of amyloid proteins in Alzheimer’s disease). This stream also regulates the alternative activation state of microglia, which is thought to be protective. The other signaling stream suppresses inflammatory reactivity and involves the repression of cytokine production and secretion [35–37]. We believe that a reduced function of TREM2 is key to the pathogenic effect of the risk variants associated with Alzheimer’s disease [22].

TREM2 is another gene known to be involved in inflammatory responses and the association found with Alzheimer’s disease corroborates the involvement of immunological pathways in this disease. This has led to an increased interest in the study of microglia (the immune cells in the brain) in these disorders. In AD, microglial cells are attracted to β-amyloid plaques, produce elevated levels of proinflammatory cytokines and reactive oxygen species, and exhibit a change in morphology [38]. Interestingly, another AD risk gene (CD33) has recently been shown to inhibit microglial uptake and clearance of β-amyloid [39].

## Therapeutic Implications

The analysis of the Mendelian families with Alzheimer’s disease led to the identification of *APP* and *Presenilin* mutations and the formulation of the amyloid cascade hypothesis [40]. Until recently, this was the overarching hypothesis for nearly all attempts to develop therapeutics for the disease. These are ongoing but have had rather limited success as yet [41, 42]. The identification of the loci from GWAs and of TREM2 by sequencing has highlighted two other pathways, which may have therapeutic potential: the first is the innate immune system and microglia activation/inflammation and the second is brain cholesterol metabolism. This latter pathway has been long suspected because of the association of Apoe with disease, but has become even clearer with the identification of *ABCA7* as an Alzheimer locus. Both of these target pathways had been suspected before, but the identification of specific genes directly involved in pathogenesis will no doubt facilitate their closer examination.

## Conclusions and Future Perspectives

We are now in an unprecedented position to study genetic variability in Mendelian and complex diseases due to the technologies developed in the last decade. The challenges ahead include the analyses of large datasets resulting from studies of cases and controls but also the integration of these analyses with family studies. The assessment of Alzheimer’s disease families will most likely reveal a small number of new fully penetrant genes, but will be essential for the identification of novel moderate risk factors.

Collecting large cohorts of well-characterized samples will be essential to accurately dissect the molecular foundations of each locus associated with AD in order to understand its genuine contributions.

## Electronic supplementary material

Below is the link to the electronic supplementary material.

ESM 1 (PDF 1224 kb)
